# Antiferromagnetic Nanoscale Bit Arrays of Magnetoelectric Cr_2_O_3_ Thin Films

**DOI:** 10.1021/acs.nanolett.4c03044

**Published:** 2024-10-10

**Authors:** Peter Rickhaus, Oleksandr V. Pylypovskyi, Gediminas Seniutinas, Vicent Borras, Paul Lehmann, Kai Wagner, Liza Žaper, Paulina J. Prusik, Pavlo Makushko, Igor Veremchuk, Tobias Kosub, René Hübner, Denis D. Sheka, Patrick Maletinsky, Denys Makarov

**Affiliations:** † Qnami AG, Hofackerstrasse 40 B, CH-4132 Muttenz, Switzerland; ‡ Helmholtz-Zentrum Dresden-Rossendorf e.V., Institute of Ion Beam Physics and Materials Research, 01328 Dresden, Germany; ¶ Kyiv Academic University, Kyiv 03142, Ukraine; § Department of Physics, University of Basel, Klingelbergstrasse 82, Basel CH-4056, Switzerland; ∥ Taras Shevchenko National University of Kyiv, 01601 Kyiv, Ukraine

**Keywords:** granular antiferromagnets, magnetoelectric Cr_2_O_3_, bit, nitrogen vacancy magnetometry, antiferromagnetic domains, magnetic memory, spintronics

## Abstract

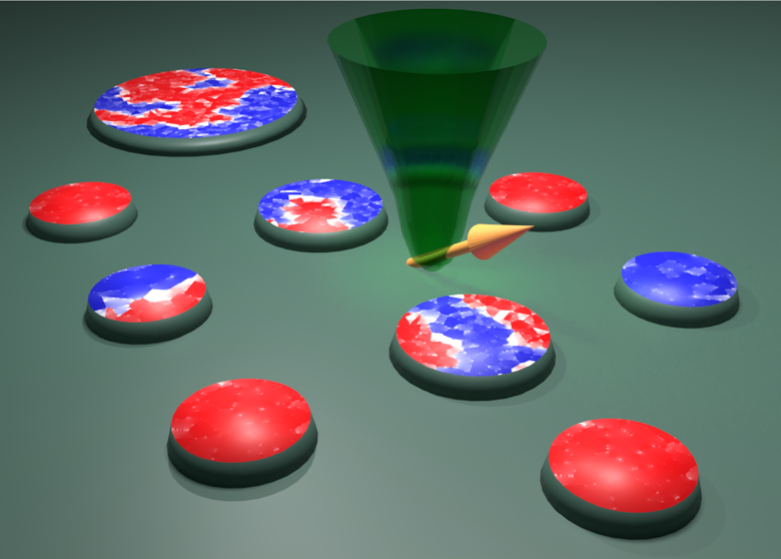

Magnetism of oxide antiferromagnets (AFMs) has been studied in single crystals and extended thin films. The properties of AFM nanostructures still remain underexplored. Here, we report on the fabrication and magnetic imaging of granular 100 nm-thick magnetoelectric Cr_2_O_3_ films patterned in circular bits with diameters ranging from 500 down to 100 nm. With the change of the lateral size, the domain structure evolves from a multidomain state for larger bits to a single domain state for the smallest bits. Based on spin–lattice simulations, we show that the physics of the domain pattern formation in granular AFM bits is primarily determined by the energy dissipation upon cooling, which results in motion and expelling of AFM domain walls of the bit. Our results provide a way toward the fabrication of single domain AFM-bit-patterned memory devices and the exploration of the interplay between AFM nanostructures and their geometric shape.

Antiferromagnetic spintronics offers major advantages over their ferromagnetic counterpart in terms of stability and density of stored information and operation speed. Recent fundamental explorations include ultrafast processes in spintronics and magnonics^1−4^ with strong focus on the physics of antiferromagnetic (AFM) materials targeting the understanding of the origin of chiral interactions,^5^ spin–orbit^6,7^ and spin-transfer torques,^8^ topological features in momentum and real space,^9,10^ finite size effects,^11,12^ strain effects,^13−15^ and the interaction of topologically nontrivial magnetic textures with lattice defects.^16−21^ There are numerous application-relevant demonstrations with AFM materials. Available proposals of AFM devices already include magnetoelectric spin–orbit (MESO) logic,^22−27^ AFM random access memory (RAM),^28−30^ magnetoelectric RAM,^31,32^ and domain-wall-based memory devices.^33^

By now, research was focused primarily on extended AFM thin films and micropatterned elements of different families of AFM materials including conducting antiferromagnets with bulk Néel spin–orbit torques like Mn_2_Au and CuMnAs,^34^ or simple oxides like NiO,^35^ Fe_2_O_3_,^19,21,36^ and Cr_2_O_3_^15,37,38^ where X-rays^39,40^ or nitrogen vacancy magnetometry^41,42^ is used for imaging. Those studies, which are performed on single crystals or extended thin films, are important for fundamental explorations, especially for domain wall physics and switching of the order parameter by external means. To explore the full application potential of these antiferromagnets, their behavior when patterned down to nanoscale dimensions must be known. To this end, there is a strong inspiration from the community working on complex oxides, including LaFeO_3_^43,44^ and BiFeO_3_.^22^ In particular, detailed characterization of sub-μm BiFeO_3_ samples^45,46^ resulted in the realization of the MESO concept,^22^ which is considered promising for prospective low-energy logic devices.

In the family of insulating AFMs, magnetoelectric Cr_2_O_3_ attracted attention due to the possibility to manipulate the magnetic order parameter magnetoelectrically^32,47,48^ and even by electric fields^14^ or spin–orbit torques only,^49^ which is paving the way toward AFM magnetoelectric RAM.^50^ Hence, being inspired by initial works on single crystals,^31^ there is active exploration of the performance of Cr_2_O_3_ thin films recently, extending studies to unconventional substrates like mica.^51^ It is established that thin films of Cr_2_O_3_ reveal flexomagnetic effects^15^ and feature finite-size effects for ultrathin films, reflected in the reduction of the transition temperature.^11^ To assess the technological relevance of Cr_2_O_3_ for high-areal-density magnetoelectronics, it is important to understand the physics of magnetic states in sub-μm bits of Cr_2_O_3_ thin films. Furthermore, a robust method to read out the magnetic state of nanoscale bits of AFM thin films should be established.

Here, we demonstrate the fabrication and measurements of nanoscale AFM Cr_2_O_3_ bits, which can accommodate an information bit-stored in the AFM order parameter. By using scanning nitrogen vacancy magnetometry (SNVM), which has proven useful to read out ferromagnetic magnetic random access memory (MRAM) bits,^52^ we experimentally observe that reducing the lateral size of individual AFM bits with 20 nm-sized grains in an array from 500 nm down to 100 nm, there is a transition from multidomain to single domain state. The physics of multidomain states in uniaxial AFM thin films of Cr_2_O_3_ is attributed to the pinning of AFM domain walls at grain boundaries. We performed spin–lattice simulations and determined the relevant intergrain coupling parameters of the order of 15% of the nominal value with a wide distribution of the intergrain exchange bonds, which allows matching the experimental and theoretically calculated AFM domain states. This is the first demonstration that Cr_2_O_3_ thin films can be used to realize nanoscale bit-patterned media. To this end, we show that Cr_2_O_3_ can be used to realize arrays of bits with a diameter of 100 nm and a period of 200 nm. Imaging of the magnetic states in the 100 nm bits of thin-film AFM Cr_2_O_3_ reveals their single domain state, as confirmed with SNVM imaging from which the map of the Néel vector is inferred using machine learning algorithms.

We deposited 200 nm-thick Cr_2_O_3_ films by reactive evaporation on *c*-cut sapphire substrates. Magnetotransport characterizations relying on the zero-offset Hall measurement scheme^32,53^ reveal the Néel temperature of the as-prepared samples to be about 301 K.^54^ The magnetic length in Cr_2_O_3_ is *l* ≈ 20 nm.^33^ By using electron beam lithography and reactive etching, thin films are patterned in square arrays of circular bits with diameters of 500 nm (period: 1000 nm), 250 nm (period: 500 nm), and 100 nm (period: 200 nm), see Figure 1a. Etching of thin films to realize bits leads to a reduction of the film thickness down to about 100 nm, as confirmed by transmission electron microscopy (TEM) imaging (Figure 1b,c). Furthermore, high-resolution TEM analysis provides access to the granular morphology of the thin films with a grain size of about 20 nm and a high crystallinity within each grain (Figure 1c). We note that individual bits are connected with a 20 nm-thick Cr_2_O_3_ layer. As Cr_2_O_3_ with a thickness of less than 30 nm is paramagnetic at room temperature,^11,55^ these bridges do not affect the interpretation of the SNVM contrast and do not contribute to the measured stray fields, as confirmed by the SNVM measurements.

**Figure 1:**
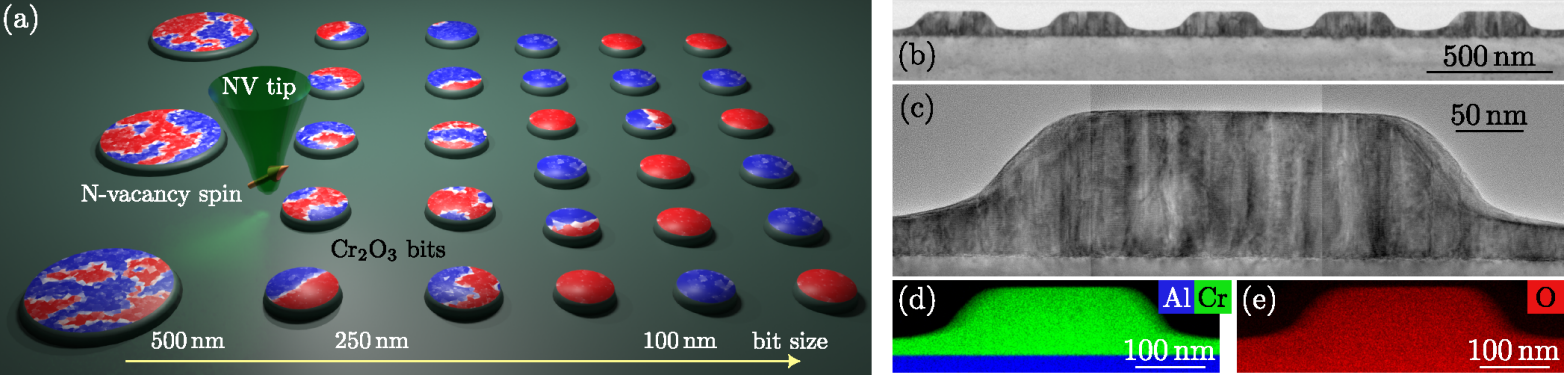
**Nanoscale arrays of antiferromagnetic bits.** (a) Schematics of the experimental setup, showing granular bits with AFM domain patterns indicated in red-blue color. The image shows that with the reduction of the bit diameter, there is a change in the domain pattern from multi- to single-domain. (b) Cross-section transmission electron microscopy image showing a regular array of Cr_2_O_3_ bits with a nominal diameter of 250 nm. (c) Stitched TEM images showing the granular structure of an individual Cr_2_O_3_ bit with a lateral grain size in the range of 20 nm. Element distribution maps from spectrum imaging analysis based on energy-dispersive X-ray spectroscopy (EDXS) for (d) aluminum and chromium and (e) oxygen.

We image the magnetic states of the bits in the arrays via SNVM.^33,41,52^ Measurements are performed on a commercialy available SNVM system (Qnami ProteusQ). The magnetic state of the samples is prepared by their annealing above the Néel temperature up to 90 °C and cooling down without magneto-electric field (zero-field cooling, ZFC procedure) or in an applied magnetic field of *B* = 550 mT and an electric field of *E* = 1.4 MV/m (field cooling, FC procedure). Field cooling allows us to prepare the sample with the Néel vector having a preferential direction. In contrast, the ZFC procedure results in a sample without a preferred orientation of the Néel vector. First, we studied the largest bits with a diameter of 500 nm (Figure 2a). The map of the magnetic stray fields ***B***nv of two 500 nm bits is shown in Figure 2b. The magnetic contrast per bit indicates that *all* measured bits of that size are in a multidomain state (see Supplementary Figure 3), where the latter is identified by the magnetic stray field, *B*nv showing a nonzero number of sign reversals (i.e., changes from red to blue imaging contrast) across the extension of the bit.

**Figure 2:**
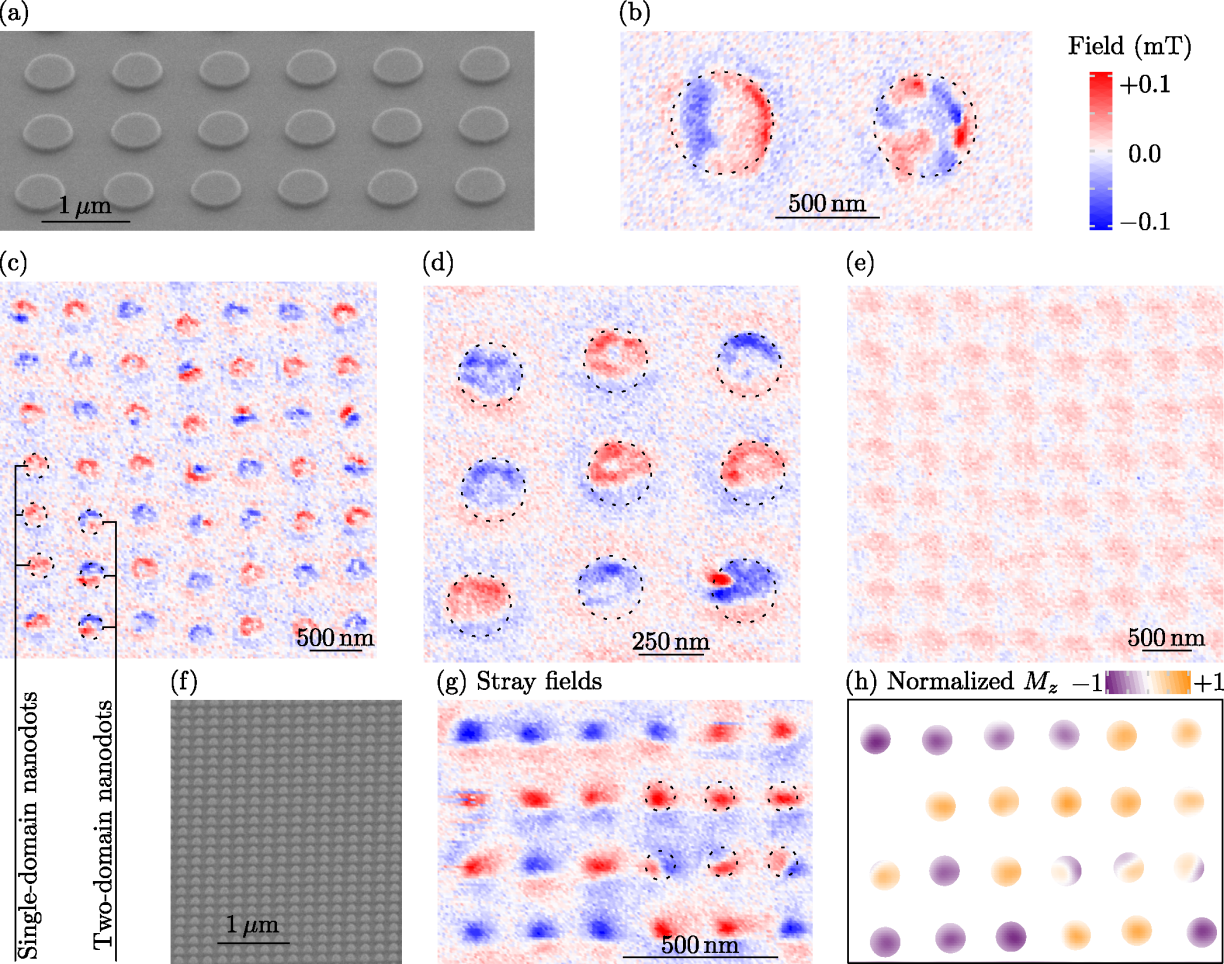
**Stray field maps and domain structure of AFM bits.** (a) Scanning electron microscopy (SEM) image of bits (500 nm in diameter) and (b) respective SNVM scan of two bits. Here, and in other panels, the dashed lines are guides to the eye and depict the selected bit position. The color bar shows the change of the stray field in the range between −0.1 and +0.1 mT. (c,d) Array of bits (250 nm in diameter) in magnetically disordered (after ZFC) and (e) ordered (after FC) states. Dashed lines show exemplary bits which are in single- or two-domain states. (f) SEM image of the bits (100 nm in diameter) and (g) respective SNVM scans. The dashed circles are guides to the eye for three single- and three two-domain bits. (h) Reconstruction of the out-of-plane magnetization from stray fields shown at the positions of the bits corresponding to panel (g). The background is blanked as a guide to the eye.

With a reduction of the bit diameter to 250 nm, some bits are still found in a two-domain state after the ZFC procedure (Figure 2c,d). We note that the number of bits with positive vs negative stray field contrast instead of the uniform one is about 30% (28 of 101 measured bits), which is in line with the assumption that the magnetic state is prepared by thermal demagnetization. Furthermore, we observe no specific pattern in the magnetic state of the bits in the array (Figure 2c and Supplementary Figure 4), which suggests that bits are magnetically decoupled. Unlike the signal from the bits, the signal between them is zero on average. The magnetic state of the bit array can be changed by magnetoelectric field cooling the sample from a temperature above the Néel temperature. A representative image of the ordered magnetic state of 200 nm-diameter bits is shown in Figure 2e. In this case, the out-of-plane magnetoelectric cooling selects a preferred orientation of the Néel vector, rendering all bits to be in the same magnetic state.

Lithographically, it is possible to fabricate magnetic bits in Cr_2_O_3_ with smaller diameters. We fabricated extended bit arrays containing bits with a diameter of 100 nm and a period of 200 nm (Figure 2f-h). Using SNVM, we are able to detect signals from individual bits in the array (Figure 2g and Supplementary Figure 5). Using the map of magnetic stray fields, we performed a reconstruction of the magnetic moment (Figure 2h). The reconstruction is done under the assumption that the magnetic texture is homogeneous along the thickness of the bit and the Néel vector is pointing along the *c*-axis. With the information that stray field maps are measured in a parallel plane at the known SNVM tip height, the magnetization can be reconstructed relying on artificial intelligence algorithms.^56−60^ The magnetic pattern shown in Figure 2h corresponds to the thermally demagnetized state. We found only about 6% of bits which may be in a multidomain state (9 of 157 measured bits). Three bits in a two-domain state are shown in Figure 2g.

Cr_2_O_3_ behaves as a two-sublattice collinear antiferromagnet. The macroscopic magnetic state of Cr_2_O_3_ is described by the Néel vector (primary order parameter) ***n*** = 0.5(***M***_1_ – ***M***_2_) defined as the difference of the unit vectors of the magnetization of the sublattices ***M***_1_ and ***M***_2_.^61^ For the case of *c*-plane samples, the direction of ***n*** can be associated with the direction of the boundary magnetization at the top surface.^62,63^ Following the approach,^60^ we modeled individual bits as granular media. In simulations, we represent each bit as a two-dimensional AFM bipartite square lattice whose dynamics is governed by the Landau–Lifshitz–Gilbert equation. Limiting the consideration of effects stemming from the exchange stiffness and anisotropy only, the static macroscopic magnetic state can be formulated within the nonlinear σ-model as for Cr_2_O_3_.^33^ The magnetic sites and associated unit vectors of the magnetic moments ***m***_*i*_, with *i* enumerating the spins, are located within a circle corresponding to the bit diameter. The grain structure is modeled via a Voronoi pattern with the average tile size equivalent to 20 nm, as in the experiment, corresponding to the grain size observed by TEM (Figure 1c). To compare experiment and simulations, we calculate the Néel texture in equilibrium. We vary the strength of exchange bonds *J*_b_ at grain boundaries according to the truncated normal distribution,^64^ with the mean value *j* and standard deviation σ measured in units of the nominal exchange strength *J*_g_. By tailoring the width of the distribution σ, the coupling between grains can vary from the strictly AFM (narrow distribution) to a mix of antiferro- and ferromagnetically coupled grains within one bit.

The magnetic state of the bit in simulations can be quantified by the measurement of the related area occupied by the Néel vectors directed upward, ⟨*n*_*↑*_⟩, in comparison with the total area of the sample. First, we examine the FC-like procedure by relaxing the bit starting from the uniform state in terms of ***n***, see Figure 3a. If *j* ≳ 0.05, the bit keeps the uniform state for all examined intergrain coupling parameters (Figure 3d). Otherwise, for 0 < *j* ≲ 0.05, the fraction of oppositely oriented domains grows with an increase of the amount of ferromagnetic exchange bonds. In the limiting case *j* < 0, a bit tends to be in a frustrated state (Figure 3c). We note that for the material parameters estimated for similar films with a thickness of 200 nm,^60^ bits are in the uniform state after the FC procedure, see also Figure 2e.

**Figure 3:**
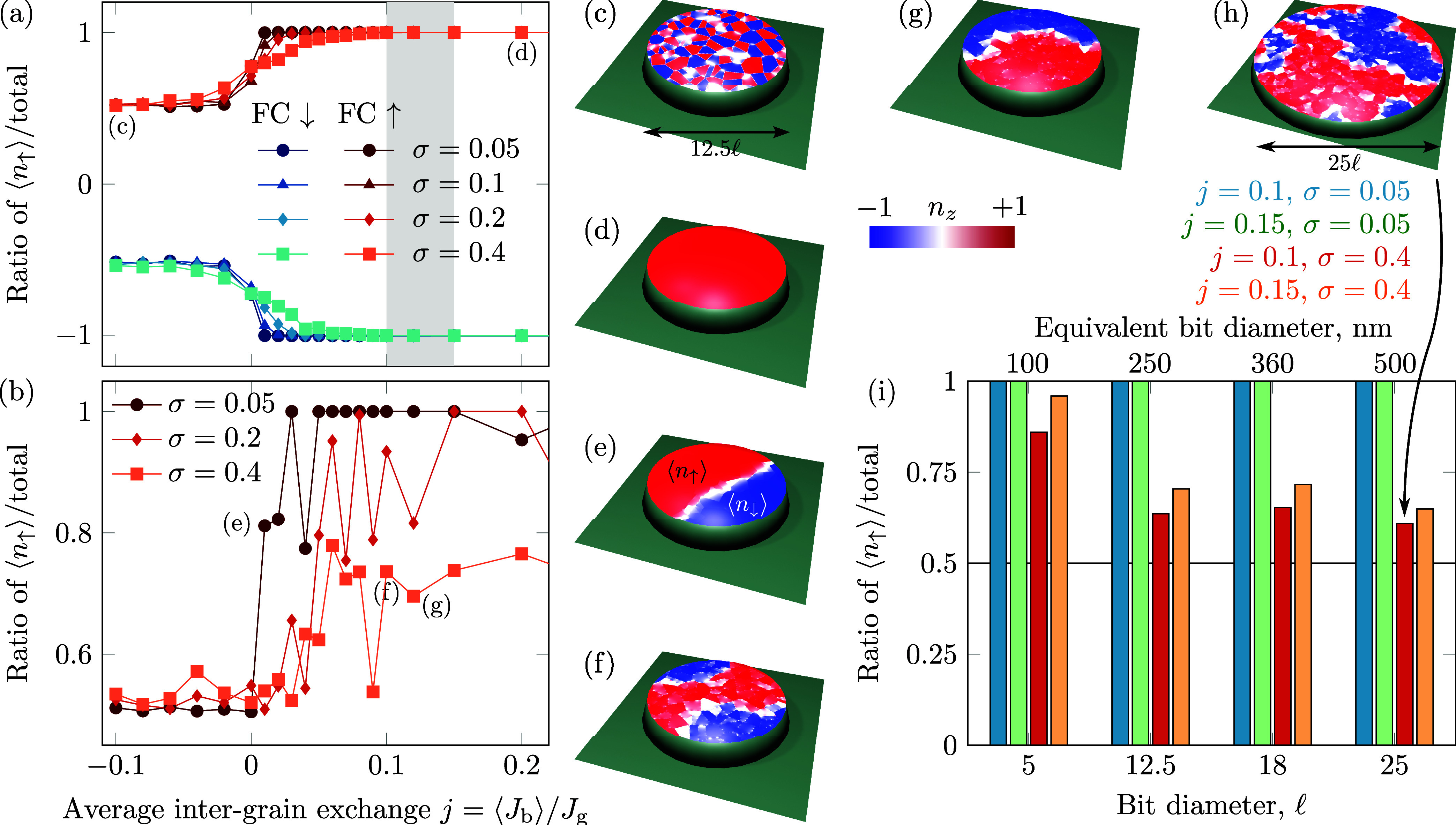
**Spin–lattice simulations of granular chromia bits.** (a) FC-like and (b) ZFC-like simulations for bits with different intergrain exchange coupling parameters (bit diameter 12.5*l*). The gray-shaded region shows the range for *j* determined in ref (60). for the case of extended thin films with a thickness of 200 nm. (c) Magnetic texture in terms of *n*_*z*_ at the bit surface for the case of *j* = −0.1 and (d) *j* = 0.2. (e–g) Examples of the evolution of the domain pattern depending on the intergrain coupling for the parameters marked in (b) with the diameter of 12.5*l* and (h) 25*l*. (i) Relative area covered by “up” domains depending on the size of bits and intergrain coupling, see detailed statistics in Supplementary Figure 13.

A ZFC-like procedure can be emulated by setting the initial state in simulations to be disordered. For a narrow distribution of exchange bonds (σ = 0.05), there is a trend for a fast saturation to the uniform state (circular symbols in Figure 3b). With larger σ, the multidomain state occurs more frequently for larger *j* (diamond symbols in Figure 3b). For a sufficiently large σ ∼ 0.4, a typical state is multidomain (square symbols in Figure 3b, see exemplary magnetic patterns in Figure 3e-h). The domain wall structure in these samples is determined by the grain size. Having grains with a characteristic size of the order of the magnetic length, it cannot accommodate the complete domain wall. Thus, the transition between domains consists of almost uniformly magnetized grains with |*n*_*z*_| being close to 0, see Figure 3f–h.

Figure 3i shows the relative area occupied by the domains oriented “up” using the ZFC-like procedure. If the distribution of the exchange bonds at grain boundaries is narrow (σ = 0.05), then the bit can be in the monodomain state independently on its size (blue and green columns in Figure 3i). In contrast, σ = 0.4 leads to a reduction of the area of the “up” domains toward half of the sample with growth of its size. At the same time, the smallest bits of 5*l* in diameter maintain almost the single-domain size independently on σ.

The calculations reveal that the described domain wall pinning behavior is a result of an interplay of the energy landscape formed by the grain boundaries and energy surplus formed by the initial paramagnetic state. For a sufficiently small bit, the energy penalty associated with a domain wall is enough to move the domain wall through all the pinning sites at boundaries to reach the uniform state independent of the pinning strength (see Figure 4 and Supplementary Figures 9–11). In larger samples, the surplus of energy is insufficient, and domain walls tend to stop being pinned at grain boundaries and usually touch the sample’s boundary.

**Figure 4:**
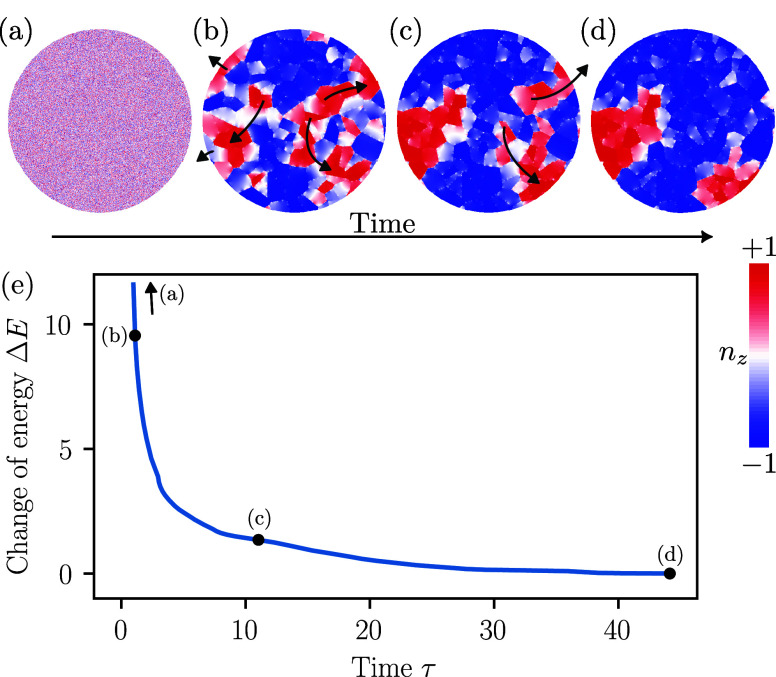
**Temporal evolution of the domain pattern (bit diameter)** 12.5*l*, *j* = 0.1, σ = 0.4). (a–d) Sequential snapshots of the magnetic state for ω_0_*t* = 0, 1.1, 11, and 44 (steady state). Dark-gray arrows indicate the direction of the domain wall motion. (e) Change of the total energy Δ*E* measured in units of *J*_g_ with time τ measured in units of inverse AFM resonance frequency. The black symbols indicate the positions corresponding to panels (a–d). See Supplementary Figure 12 for details.

To summarize, we experimentally demonstrated a possibility to (i) realize arrays of AFM Cr_2_O_3_ bits with perpendicular anisotropy, (ii) perform readout of their magnetic state by means of SNVM, and (iii) control this state using magnetoelectric cooling. We found that the bit’s lateral size is decisive for the spontaneous domain formation during ZFC procedure: while all measured bits of 500 nm diameter are in a multidomain state, only 6% of the 100 nm-sized bits are found in a two-domain state (Figure 2). These results are in a qualitative agreement with spin–lattice simulations: for a broad distribution of exchange bonds characteristic for these type of samples,^60^ smaller samples demonstrate only rare incidents of splitting in a two-domain state due to a weak domain wall pinning at grain boundaries. At the same time, equivalents of 500 nm-sized bits approach a 50/50 ratio between the oppositely oriented AFM domains as a typical state. Spin–lattice simulations show that the smallest bits are most likely to be in a single-domain state. This is in agreement with the domain imaging using SNVM, which identified only several bits in a two-domain state, with the majority being in a single-domain state.

The variation of the intergrain magnetic coupling has a strong influence on the domain structure for the *c*-plane cut sample (Figure 3i). The quality of grain boundaries can be controlled by the fabrication procedure, e.g., annealing temperature, which influences type and distribution of structural and magnetic defects.^65^ We anticipate that our findings will stimulate research on granular antiferromagnets including further miniaturization of AFM bits, consideration of other materials,^34^ and exploring spin Hall physics at nanoscale islands of Cr_2_O_3_.^32^ In particular, the shown interplay between the thermodynamical equilibrium magnetic state and size of the bit opens a way for further studies of the geometric constraints on magnetism in nanosized AFMs with in-plane anisotropy and strain effects^35,43,44,66,67^ extending them to granular materials and eas*y*-axis anisotropy. Furthermore, this work demonstrates the necessity to improve readout methods for success of using SNVM as a readout method for AFM nanoscale materials. For technological trials, all-electrical-readout for these nanoscale AFM bits should be developed.

## Supporting Information Available

Details on chromia thin film fabrication and nanopatterning; TEM characterization; details of scanning nitrogen vacancy magnetometry; description of spin–lattice simulations in granular bits; additional references (PDF)
