# Supplementary material for: Antiferromagnetic Nanoscale Bit Arrays of Magnetoelectric Cr2O3 Thin Films
Source: Nano Lett. 2024 Oct 10;24(42):13172–8. doi: 10.1021/acs.nanolett.4c03044 (PMC11503818; doi:10.1021/acs.nanolett.4c03044)
Supplement: Supplementary file 1 — nl4c03044_si_001.pdf [file nl4c03044_si_001.pdf]

Supporting information for "Antiferromagnetic nanoscale bit arrays of magnetoelectric Cr_2O_3 thin films"

Peter Rickhaus,[1,*] Oleksandr V. Pylypovskyi,[2,3,†] Gediminas Seniutinas,[1] Vicent Borras,[1] Paul Lehmann,[4] Kai Wagner,[4] Liza Žaper,[1,4] Paulina J. Prusik,[2] Pavlo Makushko,[2] Igor Veremchuk,[2] Tobias Kosub,[2] René Hübner,[2] Denis D. Sheka,[5] Patrick Maletinsky,[4] and Denys Makarov[2,‡]

[1] *Qnami AG, Hofackerstrasse 40 B, CH-4132 Muttenz, Switzerland*
[2] *Helmholtz-Zentrum Dresden-Rossendorf e.V., Institute of Ion Beam Physics and Materials Research, 01328 Dresden, Germany*
[3] *Kyiv Academic University, Kyiv 03142, Ukraine*
[4] *Department of Physics, University of Basel, Klingelbergstrasse 82, Basel CH-4056, Switzerland*
[5] *Taras Shevchenko National University of Kyiv, 01601 Kyiv, Ukraine*

CONTENTS

I. Chromia thin film fabrication and nanopatterning 1
II. TEM characterization 2
III. Scanning nitrogen vacancy magnetometry 2
IV. Spin-lattice simulations for many-grain samples 5
References 8

I. CHROMIA THIN FILM FABRICATION AND NANOPATTERNING

We prepared 200-nm-thick Cr_2O_3 thin films on the commercially available *c*-cut single-crystalline $Al_2O_3(0\,0\,0\,1)$ substrates (Crystec GmbH) with thickness of 0.5 mm by reactive evaporation of chromium at 700 °C with a background partial pressure of molecular oxygen of 10^{-5} mbar (base pressure: below 10^{-7} mbar; deposition rate of about 0.04 nm/s; source-to-sample distance: 60 cm). After the deposition of Cr_2O_3, the samples were heated up to 750 °C for several minutes to improve the surface quality of the thin films. Afterwards, the substrate was cooled down by heat dissipation through the sample mounting structure.

For lithography, the sample was cleaned in acetone and isopropanol before spin-coating the FOX-16 negative tone resist. The thickness of the resist was about 300 nm. The sample was then baked at 90°C for 3 min before being exposed in an electron beam lithography system Raith EBPG 5000+. After exposure, the sample was developed using commercially available Microposit 351 developer mixed with water in a ratio of 1:3 (developer : H_2O). The development time was 6 min at room temperature. Then the sample was etched in Sentech SI500 ICP/RIE plasma etcher with the following conditions: 400 W ICP power, 100 W RIE power, 7.5 mTorr chamber pressure, 40 sccm Cl_2 and 25 sccm Ar gas flow, etching time was set to 80 s. The sample was then cleaned in 10% hydrofluoric acid to remove any residues of the etch mask, followed by rinsing in DI water and isopropanol. We varied the current density from 9 to 16 mC/cm^2. The samples discussed in the manuscript are prepared using current density of 16 mC/cm^2.

Cr_2O_3 is an insulating antiferromagnet. To access its magnetic state, we cap the sample with a thin layer of Pt (typically, 2-3 nm) and perform magnetotransport characterization in the Hall geometry, i.e. we record changes of the transversal resistance as a function of the cooling magnetic field or temperature [1]. We demonstrated that the measured transversal resistance is sensitive to the magnetism of the underlying Cr_2O_3 layers via the Spin-Hall magnetoresistance effect [2]. Practically, we perform measurements in the so-called zero-offset Hall scheme [3, 4], which allows to reject the contribution of a parasitic longitudinal resistance to the transversal resistance channel. The Néel temperature of the as-prepared samples (i.e., extended thin film before patterning) was found to be about 301 K.

* peter.rickhaus@qnami.ch
† o.pylypovskyi@hzdr.de
‡ d.makarov@hzdr.de

II. TEM CHARACTERIZATION

Cross-sectional bright-field transmission electron microscopy (TEM) and high-resolution TEM (HRTEM) analyses were performed using an image-C_s-corrected Titan 80-300 microscope (FEI, Field Electron and Ion Company) operated at an accelerating voltage of 300 kV. High-angle annular dark-field scanning TEM (STEM) imaging and spectrum imaging analysis based on energy-dispersive X-ray spectroscopy (EDXS) were done with a Talos F200X microscope (FEI) operated at 200 kV. Prior to (S)TEM analysis, the specimen was mounted in a double-tilt high-visibility low-background holder and placed for 8 s into a Model 1020 Plasma Cleaner (Fischione) to remove potential contaminations. Cross-sectional preparation of the TEM lamella was done by in situ lift-out using a Helios 5 CX focused ion beam (FIB) device (Thermo Fisher). To protect the sample surface, a carbon cap layer was deposited beginning with electron-beam-assisted and subsequently followed by Ga-FIB-assisted precursor decomposition. Afterwards, the TEM lamella was prepared using a 30-keV Ga-FIB with adapted currents. Its transfer to a 3-post copper lift-out grid (Omniprobe) was done with an EasyLift EX nanomanipulator (Thermo Fisher). To minimize sidewall damage, Ga ions with only 5-keV energy were used for final thinning of the TEM lamella to electron transparency.

A single-domain state of Cr_2O_3 crystals is characteristic for high-quality single crystals [5]. In granular thin films, the thermodynamically stable state includes multiple domains pinned at grain boundaries [6, 7]. The observed domain structure is created by antiphase domain walls in the Néel vector instead of the change of spin structure only at the surface of the sample. The peculiar properties of the c-plane crystallographic termination of Cr_2O_3 allow to consider it as a monolayer of ferromagnetically ordered spins, which belong to one magnetic sublattice [8]. The stray fields are generated at noncollinear magnetic textures like domain walls [5].

As the sample consists of rather tall bits, high-resolution atomic force microscopy measurements at the local scale of individual bits are challenging. Therefore, the roughness of the top surface of the samples at the local scale can be addressed by TEM (Supplementary Fig. 1). The relevant cross-sectional images are shown in Figure 1b,c . We see an increase of the surface roughness at the side facets of the bit. However, the top surface of the sample remains smooth, even after lithographic patterning. Still, the interior of the bits consists of individual grains with a size of about 20 nm. Our current understanding is that antiferromagnetic domain walls do pin at grain boundaries but not at the surface roughness. Furthermore, we note that this expectation is in line with the commonly accepted model that the magnetic texture in c-plane Cr_2O_3 is roughness-insensitive [9]. Furthermore, we note that the comparison of atomic force microscopy and scanning nitrogen vacancy magnetometry maps measured on the same bits does not reveal a correspondence between the antiferromagnetic domains and surface topography, see Supplementary Fig. 2.

III. SCANNING NITROGEN VACANCY MAGNETOMETRY

Scanning nitrogen vacancy magnetometry (SNVM) [10] was performed under ambient conditions at about 22° C on the Qnami ProteusQ system at remanence, without magnetic and/or electric field applied to the sample. The sensor tips (Qnami Quantilever MX+) are fabricated from single-crystalline ⟨100⟩-oriented diamond that was implanted with ^{14}N ions at 12 keV and annealed to form nitrogen vacancy centers. After lithographic fabrication, the resulting diamond scanning probe contains a single nitrogen vacancy center, located at the tip of a parabolic pillar, and provides a spatial resolution of approximately 50 nm [11] and typical sensitivities of approximately $3\,\mu\mathrm{T}/\sqrt{\mathrm{Hz}}$ [11]. Mapping of magnetic stray fields is performed using continuous-wave optically detected magnetic resonance (CW-ODMR) imaging [10]. For a sign-sensitive measurement of stray fields, a small bias magnetic field of about 50 Oe was applied during all measurements. The single-pixel integration time for the presented measurements range from 0.6 s to 7 s.

To prepare the magnetic state, we apply electric field using a macroscopic capacitor-like structure. The sample is sandwiched between two metallic electrodes. As top and bottom electric contacts ,we use centimetre-sized electrodes prepared by evaporating gold films on glass slides. The electrodes are not in direct contact with the sample. The sample is mounted with the patterned side fasing up between two mica sheets and then two gold electrodes. The mica sheets are needed to isolate the smaple from the gold, because we had gold coating the samples in the process without mica. We apply a voltage of 709 V using a high-voltage power supply. Since the sample thickness (thin film of Cr_2O_3 on Al_2O_3 substrate) is about 0.5 mm, the average electric field between the electrodes is about 1.4 MV/m. We note that the field distribution inside the sample, and therefore the field in the chromia thin film, depends on the thickness and relative permeabilities of all layers in the stack. With our setup we cannot control the leak current. Still, the leak current, if present, should be smaller than a 1 mA, which is a compliance current of the power supply.

The electric field was applied perpendicular to the surface of the sample and out of the structured (top) surface of the sample. The magnetic field was applied in the same direction, so the field cooling was done in parallel magnetoelectric field oriented perpendicular to the surface of the sample. To prepare the single domain state, the process was as follows:

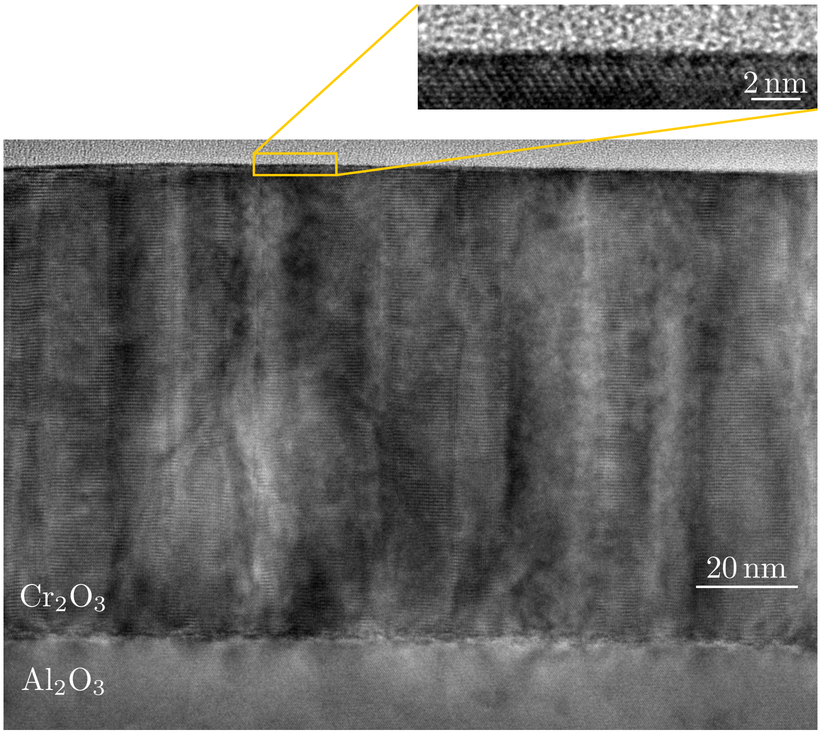

Supplementary Fig. 1. TEM image taken from the central part of a bit. The inset shows the top surface with a roughness of less than 1 nm.

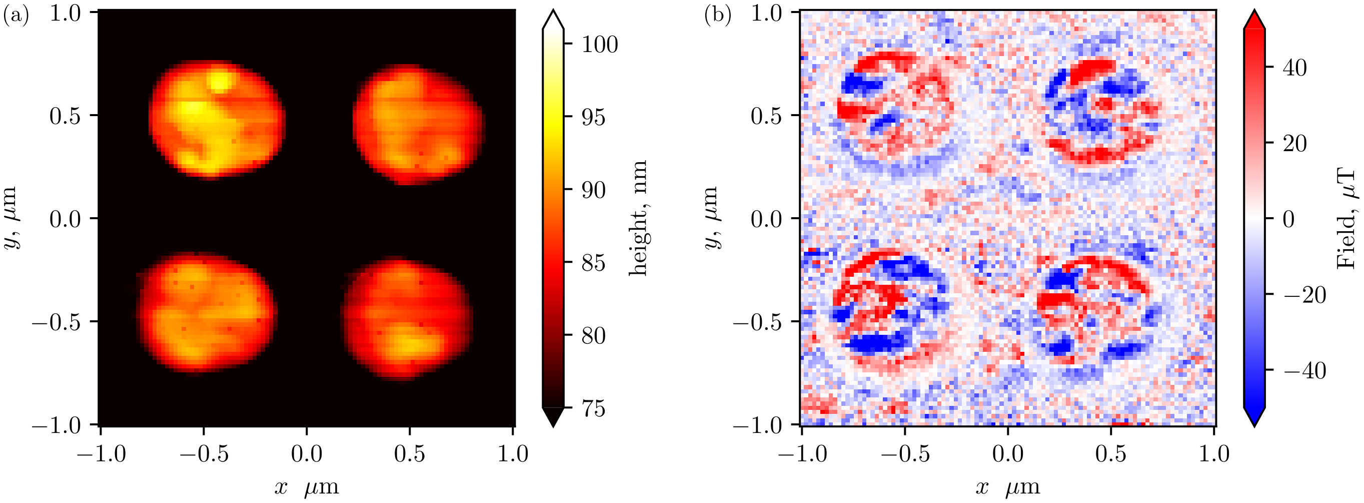

Supplementary Fig. 2. Comparison of (a) the surface topography obtained by means of atomic force microscopy and (b) scanning nitrogen vacancy magnetometry stray field maps measured on the same bits. We note that there is no correlation between the topography and magnetic domains.

1. Expose the sample to an external magnetic field of 550 mT at room temperature, which is about 295 K. The magnetic field was pointing up out-of-plane.

2. Expose the sample to an external electric field of -709 V with respect to the bottom electrode (at room temperature). The electric field is pointing up as well.

3. Heat the sample to 355 K, which is above the Néel temperature. The heat was applied with a heatgun. The temperature was monitored with a thermistor.

4. Cool the sample through the Néel temperature (of about 307 K) to room temperature with both electric and

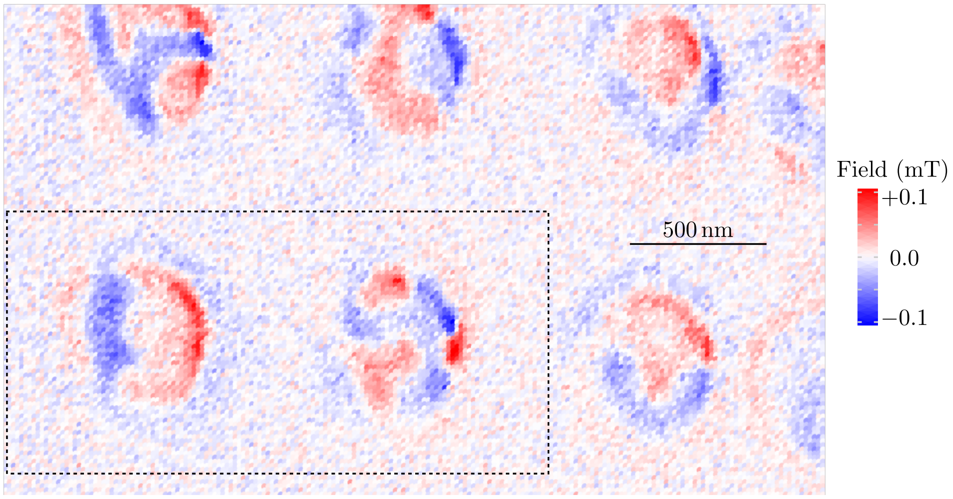

Supplementary Fig. 3. Stray field map for bits of 500 nm in diameter. The dotted area indicates the region shown in Fig. 2b of the main text.

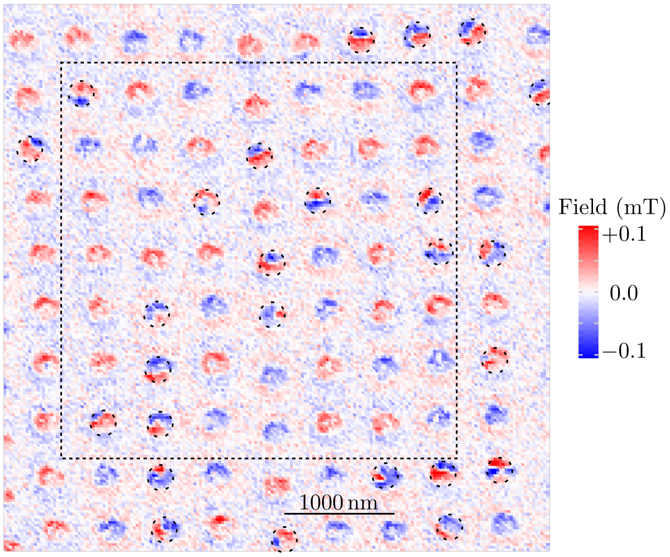

Supplementary Fig. 4. Stray field map for bits of 250 nm in diameter. The rectangular dotted area indicates the region shown in Fig. 2c of the main text. The round dashed lines indicate bits in a multidomain state.

magnetic fields applied.

5. Switch off the magnetoelectric fields and start SNVM measurements at room temperature.

The current model of the c-cut of Cr_2O_3 established by Belashchenko [8] suggests that magnetic textures in these samples should be roughness-insensitive. Thus, the experimentally observed domain structure is created by antiphase domain walls in the Néel vector instead of the change of spin structure only at the surface of the sample. The magnetic stray fields, which are measured by the SNVM microscope, are generated at noncollinear magnetic textures like domain walls [5]. A typical strength of stray fields generated by a single domain wall in Cr_2O_3 is in the range of $50 \ldots 100\,\mu$T [5, 6]. Topographical steps on the surface of chromia samples with the thickness of about 200 nm generate stray fields in the range of $10 \ldots 20\,\mu$T [5, 6].

The reconstruction of the magnetic texture is done under assumption that the magnetic texture is homogeneous along the thickness of the bit and the Néel vector is pointing out-of-plane (parallel to the c axis). With the stray field maps obtained with the nitrogen vacancy center in a parallel plane, magnetization can be reconstructed relying on the machine learning algorithm presented Dubois *et al.* [12]. In this reconstruction, we iterated over 1000 epochs

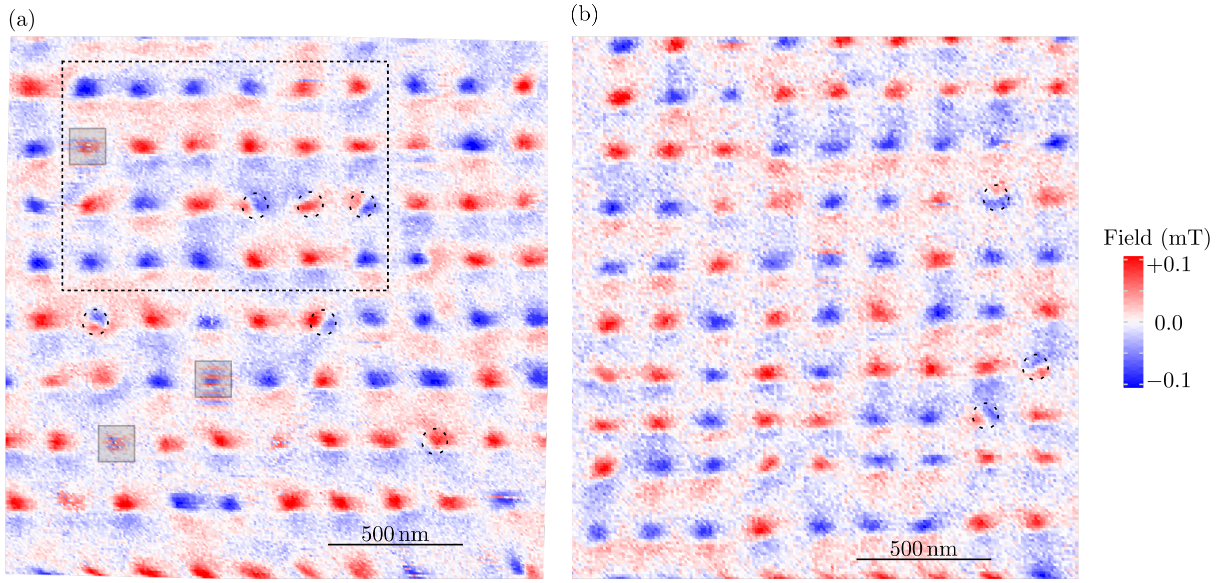

Supplementary Fig. 5. Stray field maps for bits of 100 nm in diameter measured in two different regions on the sample. The rectangular dotted area indicates the region shown in Fig. 2g of the main text. The round dashed lines indicate bits in a multidomain state. The gray semitransparent squares indicate bits within the field of view, which are excluded from consideration as their state cannot be identified.

and assumed the nitrogen vacancy center was at 35 ± 5 nm from the sample and with a polar and azimuthal angles of $54.5°$ and $90°$, respectively.

IV. SPIN-LATTICE SIMULATIONS FOR MANY-GRAIN SAMPLES

In simulations, the magnetic energy of film is given by the anisotropic Heisenberg Hamiltonian

$$\mathcal{H} = -\frac{S^2}{2}\sum_{i,k} J_{ik}\boldsymbol{m}_i\cdot\boldsymbol{m}_k - \frac{KS^2}{2}\sum_i m_{iz}^2, \tag{1}$$

where J_{ik} is the exchange integral between the i-th and k-th spins, index k runs over the nearest neighbors of i, S is the spin length, $\boldsymbol{m}_i = \{m_{ix}, m_{iy}, m_{iz}\}$ represents the unit vector of the magnetic moment for the i-th site and K is the coefficient of the easy axis anisotropy. Equilibrium magnetic textures are addressed by solving the Landau–Lifshitz–Gilibert equation

$$\frac{d\boldsymbol{m}_i}{dt} = \frac{1}{\hbar S}\boldsymbol{m}_i \times \frac{\partial\mathcal{H}}{\partial\boldsymbol{m}_i} + \alpha_{\text{G}}\boldsymbol{m}_i \times \frac{d\boldsymbol{m}_i}{dt}, \quad i = \overline{1,\mathcal{N}}, \tag{2}$$

with $\hbar$ being the Planck constant, α_{G} being the Gilbert relaxation parameter, and $\mathcal{N}$ being the total number of spins. The set of equations (2) is solved using the spin-lattice simulator SLaSi with graphics processing unit (GPU) acceleration [13]. Following the procedure described in [7], we set $J_{ik} = J_{\text{g}} = -2.34\times10^{-20}$ J as the nominal exchange integral within each grain, $K = 3.656\times10^{-24}$ J as the coefficient of the easy axis anisotropy, $S = 1/2$, and $\alpha_{\text{G}} = 1$. The magnetic length in units of the lattice constant a is $\ell = a\sqrt{|J_{\text{g}}/K_{\text{g}}|} = 80a$ to assure a high resolution of the sample's state. The mesh size varies from 400×400 to 2000×2000 lattice sites, which are located within a circle of the maximal available diameter corresponding to the size of a magnetic bit. We note that this approach is valid to analyze static distributions of the Néel vector in Cr_2O_3 on spatial scales, which are much larger than the size of its crystallographic unit cell. In the main text, we define $E = \mathcal{H}/J_{\text{g}}$ as the dimensionless energy and $\tau = \omega_0 t$ as the dimensionless time with $\omega_0 = K/(\hbar S)$ being the frequency of antiferromagnetic resonance.

The grain structure is defined by a Voronoi pattern, which determines the exchange bonds that are subjected to changes because of the grain boundary. The inter-grain coupling is simulated as a truncated normal distribution of the exchange bonds J_{ik} with the mean value $j = \langle J_{ik}\rangle/J_{\text{g}}$ and standard deviation $\sigma = 0.01\ldots0.4$ in units of J_{g} (Supplementary Fig. 6). For a bipartite square lattice, the antiferromagnetic unit cell is represented by neighboring

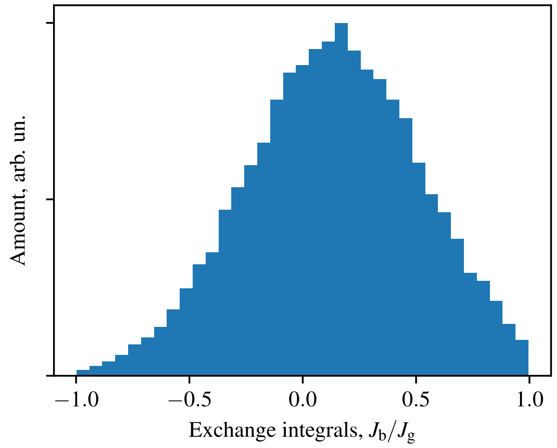

Supplementary Fig. 6. An exemplary distribution of exchange bonds at grain boundaries for $j = 0.15$ and $\sigma = 0.4$.

spins arranged in a square. For the ν-th square with magnetic moments labeled as $\boldsymbol{A}_\nu$, $\boldsymbol{B}_\nu$, $\boldsymbol{C}_\nu$, $\boldsymbol{D}_\nu$ in the counter-clockwise order, the discrete Néel order parameter is defined as $\boldsymbol{n}_\nu = \boldsymbol{A}_\nu - \boldsymbol{B}_\nu + \boldsymbol{C}_\nu - \boldsymbol{D}_\nu$ [14, 15].

Supplementary Fig. 7 shows exemplary results of simulations for the bits with diameters of 5ℓ and 12.5ℓ (i.e., equivalent to 100 nm and 250 nm, respectively). The energy drops very fast at the very beginning when the ordering from the random texture to an antiferromagnetic one happens. This is followed with high values of the dynamics rate measured as $\max|d\boldsymbol{m}/dt|$. When it's value drops to be of the order of 1, only domain wall jumps between grains are observed.

Supplementary Fig. 8 shows the evolution of the domain wall structure with the inter-grain coupling. For a weak and more spatially uniform exchange coupling (Supplementary Fig. 8a), the domain wall is almost always represented by the grain boundary itself, while the neighboring grains have the opposite values of n_z close to saturation. In this case, the thickness of the domain walls is primarily determined by the grain size and is about ℓ. Larger and spatially distributed coupling between grains leads to the appearance of grains which have a substantial amount of spins lying in the hard plane or at least strongly tilted from the easy axis. This state appears because of the average grain size being of about ℓ, which forces each grain to be in the magnetically uniform state. The average orientation of spins in this case is determined by the magnetic orientation of the surrounding grains. This is especially well pronounced in Supplementary Fig. 8d, where a lot of grains in the vicinity of the domain wall have a weak blue-to-red color gradient indicating value of n_z. We note that in case of a narrow distribution of exchange bonds and large enough j, the bulk-like domain wall can be pinned, see Supplementary Fig. 8e. The pinning strength of this state on grain boundaries is very small and such textures are rarely observed in simulations.

A possible equilibrium magnetic state of the bit is determined by the combination of its diameter and inter-grain coupling. Exemplary pictures of the temporal evolution in zero-field-cooling-like (ZFC-like) procedure are shown in Supplementary Figs. 9–12. In all cases, the initial state shown in Supplementary Figs. 9a–12a is a random orientation of spins, which are characteristic of a thermally demagnetized bit. A narrow distribution of exchange bonds creates a shallow energy landscape for domain walls. Usually, they do not pin at grain boundaries because of a substantial excess of energy and leave the sample. During their dynamics, the domain wall shape is almost not disturbed by the presence of grains. These states are shown in Supplementary Figs. 9b–f and 10b–l, were the granular structure is not pronounced in the equilibrium magnetic texture but occasionally deforms the domain wall during its movement (Supplementary Fig. 10d). Thus, independent of the bit size, a narrow distribution of exchange bonds leads to a single-domain state, see also Fig. 3i of the main text.

Magnetic dynamics for the bits with a wide distribution of exchange bonds is substantially different. Starting from the random orientation of spins, a small enough sample finally comes to a single-domain state (Supplementary Fig. 11). The Néel texture is represented by almost uniformly ordered grains and domain motion is represented by a reversal of the order parameter within the grain, which is observed for both, small bit of 5ℓ in diameter (Supplementary Fig. 11b–f) and larger bits of 12.5ℓ in diameter (Supplementary Fig. 12b–l). The difference in lateral sizes of these two bits influences the energy dissipation process. For a small bit (Supplementary Fig. 11b–l), the initial energy pumped into the system by temperature (the initial random state) is enough to push the domain wall through the whole sample to set it into a single-domain state. Larger bits (Supplementary Fig. 12b–l) have the same areal energy density at the beginning as smaller bits. Therefore, the thermal energy can be insufficient to move the domain wall over a large distance. Hence, the domain wall is affected by pinning at grain boundaries. Finally, this leads to a formation of several large almost uniformly ordered regions contacting with boundaries (Supplementary Fig. 12l).

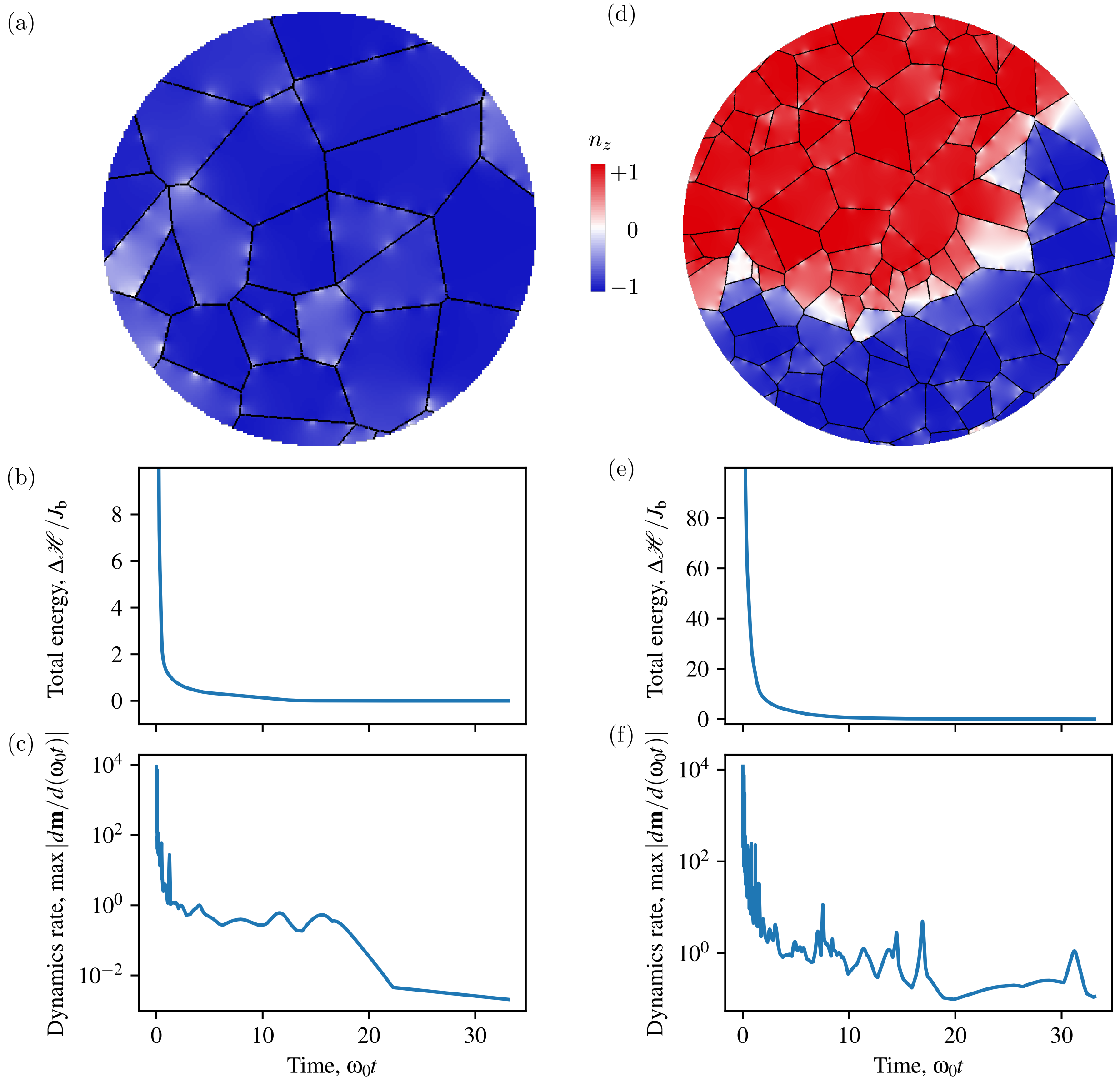

Supplementary Fig. 7. **Simulations of granular bits.** (a) Granular structure of a bit with a diameter of 5ℓ (100 nm in equivalent) with $j = 0.15$ and $\sigma = 0.4$. The orientation of the Néel vector $\boldsymbol{n}$ is shown with color, grain boundaries are shown by black lines. (b) Change of the energy with time and (c) dynamics rate for the texture shown in panel (a). (d–f) Same for the bit with a diameter of 12.5ℓ (250 nm in equivalent).

The final dynamics is related to the adjustment of the orientation of $\boldsymbol{n}$ within certain grains.

Fig. 13 summarizes the statistical analysis of the single- or multidomain behavior of granular bits after the ZFC-like procedure extending Fig. 3i in the main text. The data is grouped by different bit diameters corresponding to the samples in experiment. Each dataset presented by a box plot contains 10 simulations started from different random orientations of magnetic moments. All simulations with inter-grain exchange coupling of $j = 0.1$ and $j = 0.15$ with a narrow distribution $\sigma = 0.05$ result in a uniform state independent of the disk diameter, which makes the box plot being degenerated to a line. These points are highlighted by blue and green symbols. A broad distribution of exchange bonds, $\sigma = 0.4$, drastically changes the picture. For a small bit diameter of 5ℓ, a domain wall, if present, is located approximately to the center of the bit. We found two such cases for $j = 0.1$ and none for $j = 0.15$, which is reflected in a tall orange box in Fig. 13 for a bit of the smallest diameter. For bits of lager diameters, the median area occupied by a domain of a certain direction is below 70% and approaches 50% for the largest samples investigated in the simulations. At the same time, only for bits with the diameter of 25ℓ, the single-domain state has not been observed. This is indicated by whiskers ending at the level of about 80% of areal filling of the largest domain; for

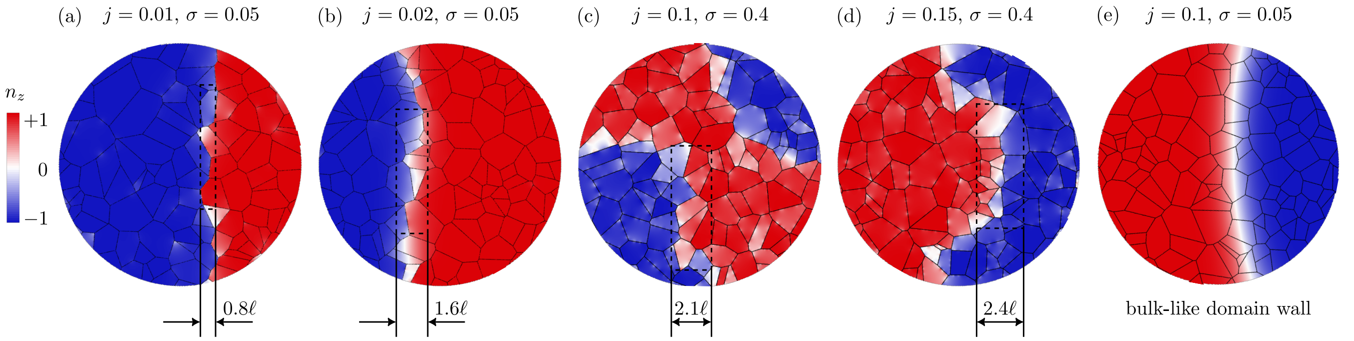

Supplementary Fig. 8. **Domain walls in bits with different inter-grain coupling.** The simulated samples have a diameter of 12.5ℓ. The dashed area shows the domain wall region for which its width is estimated. Grain boundaries are shown by thin black lines. We note that the state (e) is rarely observed in simulations because of a weak pinning for the selected inter-grain coupling.

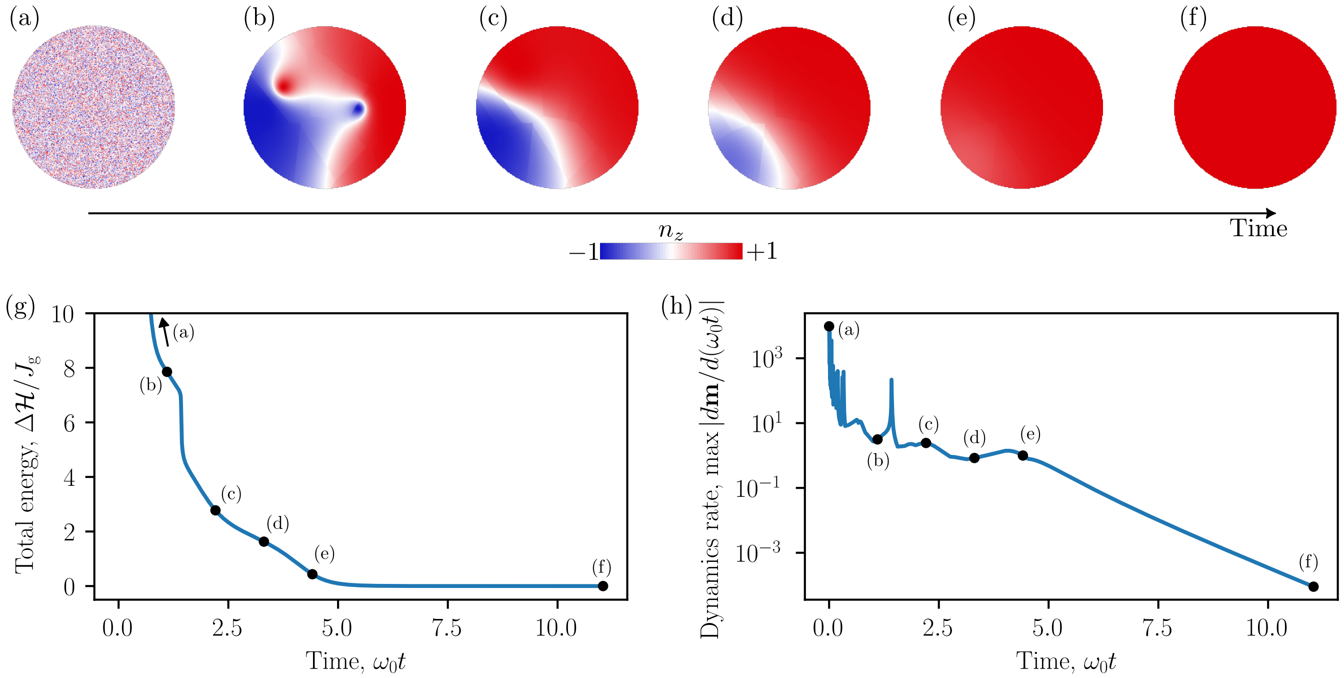

Supplementary Fig. 9. **Magnetic dynamics in ZFC-like simulations (bit diameter 5ℓ, grain coupling is given by $j = 0.1$ and $\sigma = 0.05$).** (a–f) Sequential magnetic states colored according to the value of n_z. (g) Change of the total energy with time. The black symbols show the time positions corresponding to panels (a–f). (h) Dynamics rate. Notations are the same as in panel (g).

bits of smaller diameters the top whiskers end almost at the 100% areal filling indicating a possibility to get the single-domain state in the ZFC-like procedure.

[1] T. Kosub, M. Kopte, F. Radu, O. G. Schmidt, and D. Makarov, All-electric access to the magnetic-field-invariant magnetization of antiferromagnets, Physical Review Letters **115**, 097201 (2015).
[2] R. Schlitz, T. Kosub, A. Thomas, S. Fabretti, K. Nielsch, D. Makarov, and S. T. B. Goennenwein, Evolution of the spin Hall magnetoresistance in Cr_2O_3/Pt bilayers close to the Néel temperature, Applied Physics Letters **112**, 132401 (2018).
[3] Hzdr innovation gmbh products, tensormeter measurement device. `https://hzdr-innovation.de/en/products/tensormeter/` (accessed 2024-09-20).
[4] T. Kosub, M. Kopte, R. Hühne, P. Appel, B. Shields, P. Maletinsky, R. Hübner, M. O. Liedke, J. Fassbender, O. G.

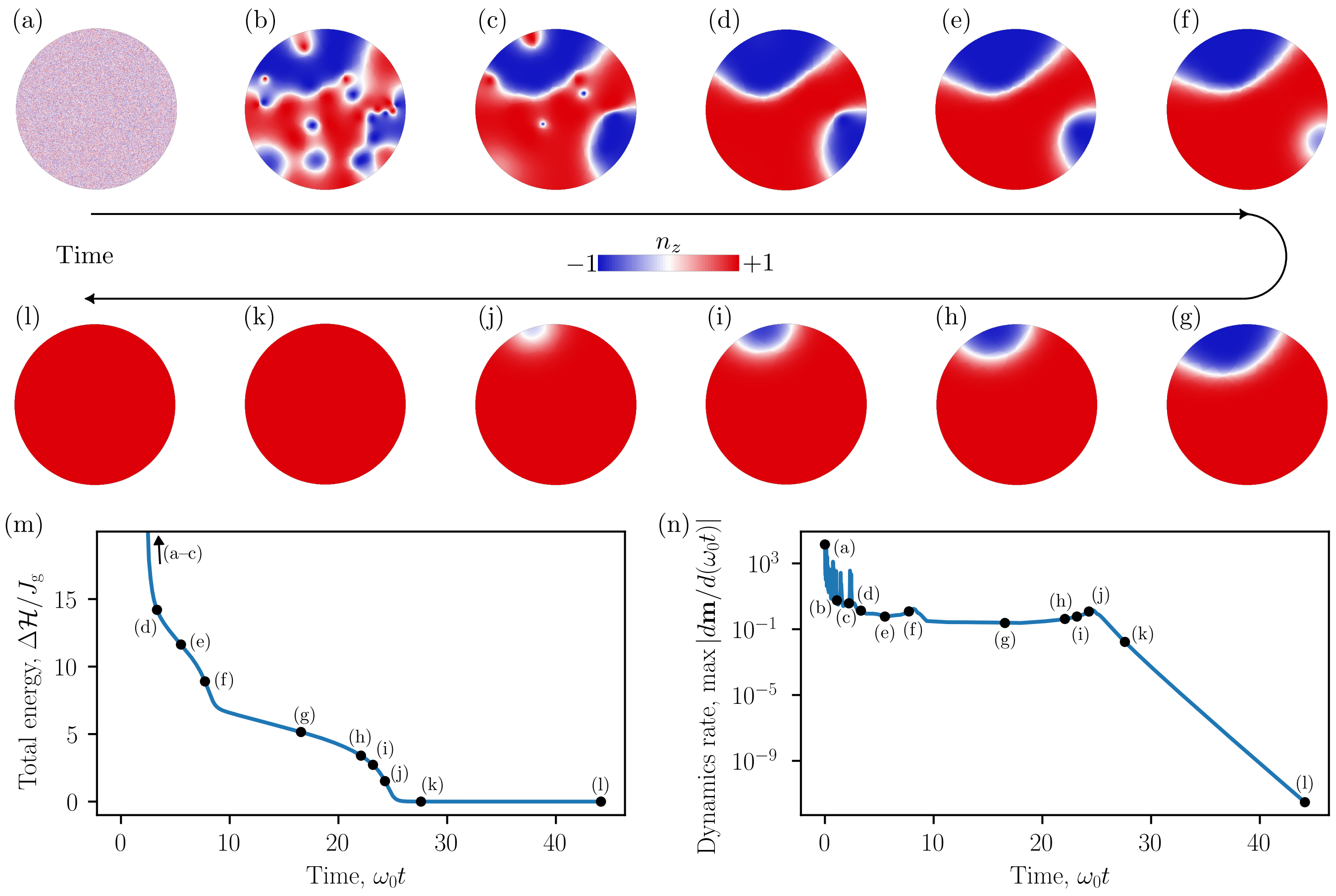

Supplementary Fig. 10. **Magnetic dynamics in ZFC-like simulations (bit diameter 12.5ℓ, grain coupling is given by $j = 0.1$ and $\sigma = 0.05$).** (a–l) Sequential magnetic states colored according to the value of n_z. (m) Change of the total energy with time. The black symbols show the time positions corresponding to panels (a–l). (n) Dynamics rate. Notations are the same as in panel (m).

Schmidt, and D. Makarov, Purely antiferromagnetic magnetoelectric random access memory, Nature Communications **8**, 13985 (2017).

[5] N. Hedrich, K. Wagner, O. V. Pylypovskyi, B. J. Shields, T. Kosub, D. D. Sheka, D. Makarov, and P. Maletinsky, Nanoscale mechanics of antiferromagnetic domain walls, Nature Physics **17**, 574 (2021).

[6] P. Appel, B. J. Shields, T. Kosub, N. Hedrich, R. Hübner, J. Faßbender, D. Makarov, and P. Maletinsky, Nanomagnetism of magnetoelectric granular thin-film antiferromagnets, Nano Letters **19**, 1682 (2019).

[7] O. V. Pylypovskyi, N. Hedrich, A. V. Tomilo, T. Kosub, K. Wagner, R. Hübner, B. Shields, D. D. Sheka, J. Fassbender, P. Maletinsky, and D. Makarov, Interaction of domain walls with grain boundaries in uniaxial insulating antiferromagnets, Physical Review Applied **20**, 014020 (2023).

[8] K. D. Belashchenko, Equilibrium magnetization at the boundary of a magnetoelectric antiferromagnet, Physical Review Letters **105**, 147204 (2010).

[9] S. F. Weber, A. Urru, S. Bhowal, C. Ederer, and N. A. Spaldin, Surface magnetization in antiferromagnets: Classification, example materials, and relation to magnetoelectric responses, Physical Review X **14**, 021033 (2024).

[10] L. Rondin, J.-P. Tetienne, T. Hingant, J.-F. Roch, P. Maletinsky, and V. Jacques, Magnetometry with nitrogen-vacancy defects in diamond, Reports on Progress in Physics **77**, 056503 (2014).

[11] N. Hedrich, D. Rohner, M. Batzer, P. Maletinsky, and B. J. Shields, Parabolic diamond scanning probes for single-spin magnetic field imaging, Physical Review Applied **14**, 064007 (2020).

[12] A. Dubois, D. Broadway, A. Stark, M. Tschudin, A. Healey, S. Huber, J.-P. Tetienne, E. Greplova, and P. Maletinsky, Untrained physically informed neural network for image reconstruction of magnetic field sources, Physical Review Applied **18**, 064076 (2022).

[13] `SLaSi` spin–lattice simulations package `http://slasi.knu.ua` (accessed 2024-09-20).

[14] S. Komineas and N. Papanicolaou, Vortex dynamics in two-dimensional antiferromagnets, Nonlinearity **11**, 265 (1998).

[15] S. Komineas and N. Papanicolaou, Traveling skyrmions in chiral antiferromagnets, SciPost Physics **8**, 86 (2020).

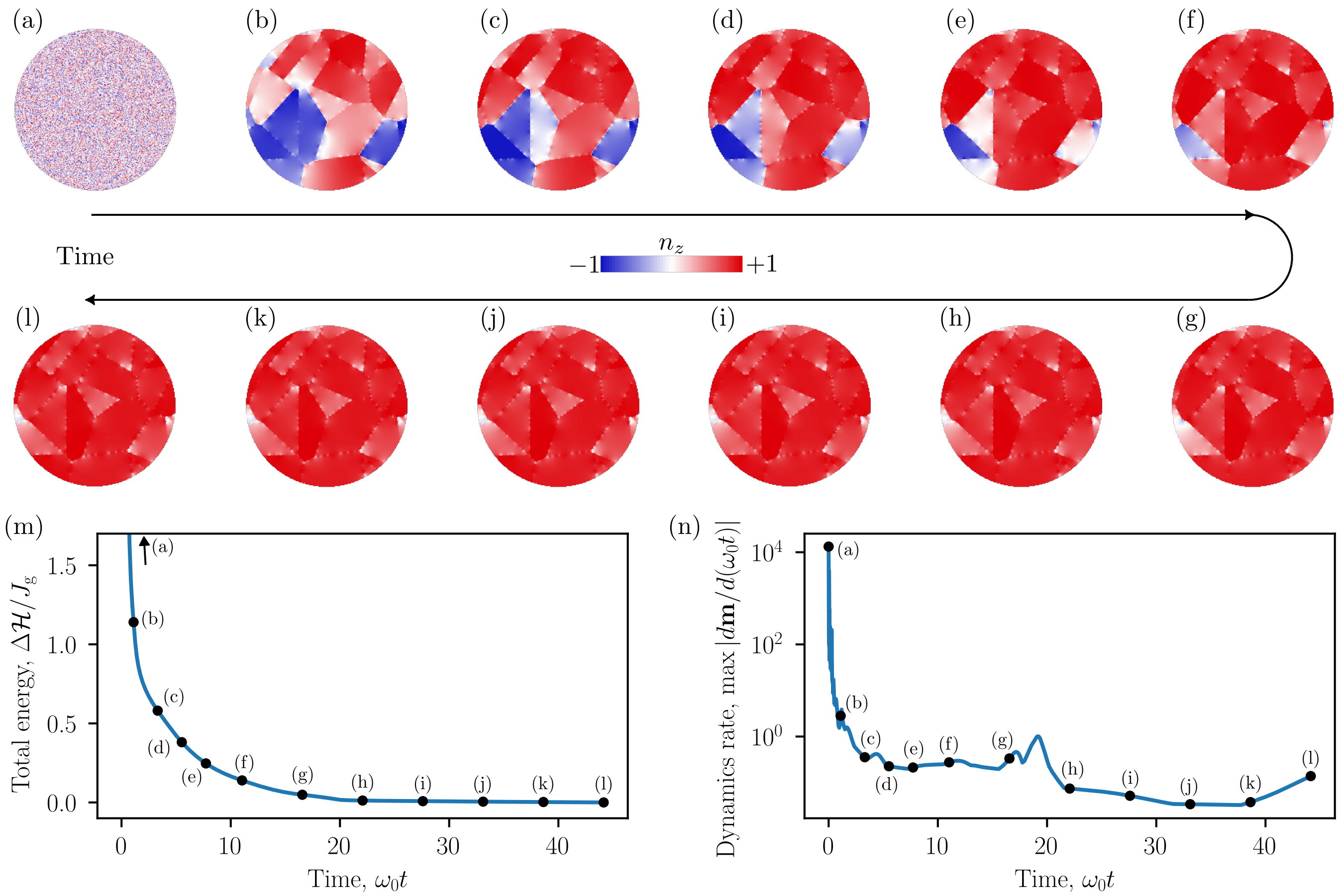

Supplementary Fig. 11. **Magnetic dynamics in ZFC-like simulations (bit diameter 5ℓ, grain coupling is given by $j = 0.1$ and $\sigma = 0.4$).** (a–l) Sequential magnetic states colored according to the value of n_z. (m) Change of the total energy with time. The black symbols show the time positions corresponding to panels (a–l). (n) Dynamics rate. Notations are the same as in panel (m).

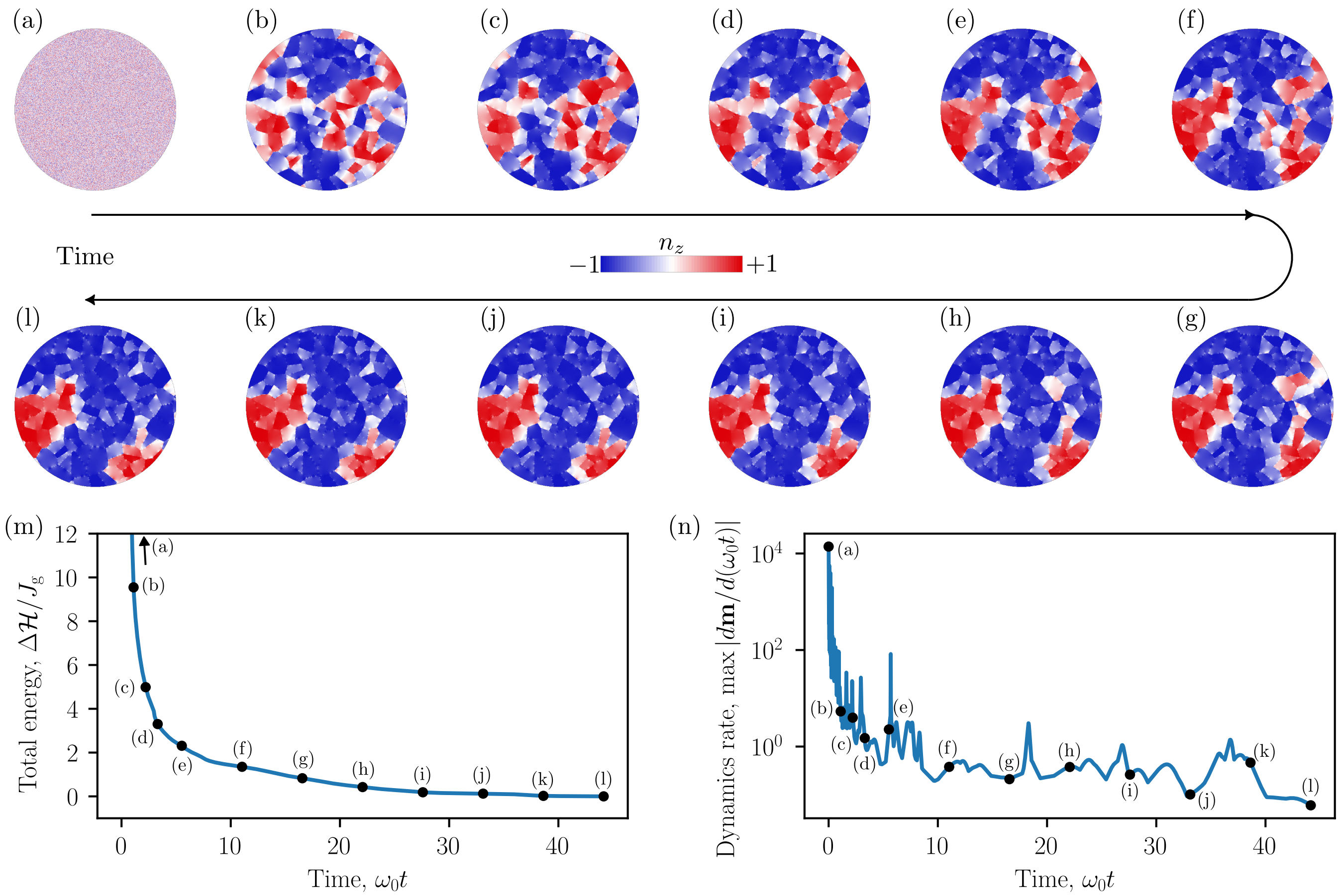

Supplementary Fig. 12. **Magnetic dynamics in ZFC-like simulations (bit diameter 12.5ℓ, grain coupling is given by $j = 0.1$ and $\sigma = 0.4$).** (a–l) Sequential magnetic states colored according to the value of n_z. (m) Change of the total energy with time. The black symbols show the time positions corresponding to panels (a–l). (n) Dynamics rate. Notations are the same as in panel (m).

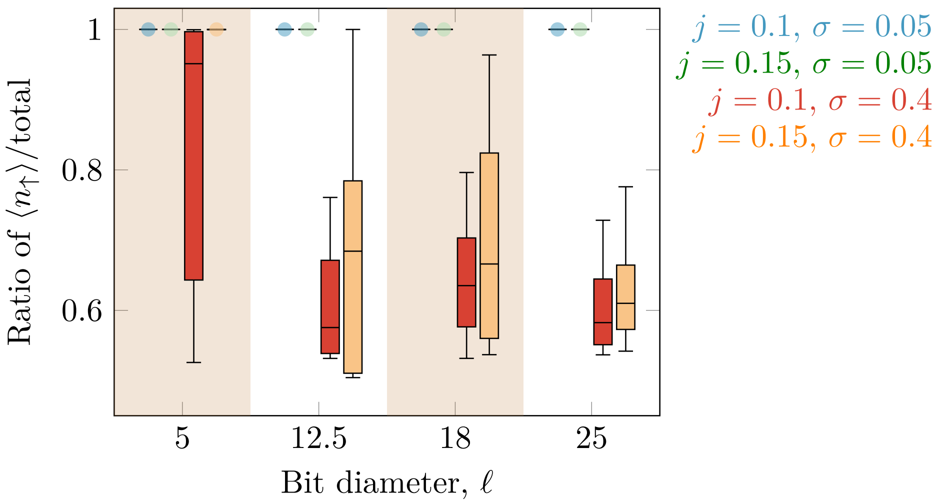

Supplementary Fig. 13. **Statistics of magnetic states in the ZFC-like procedure.** In each box, the horizontal line indicates median (c.f. with the mean value shown in Fig. 3i of the main text), box bottom and upper limits indicate lower and upper boundaries of the second and third quartile, respectively, and whiskers indicates boundaries of the first and fourth quartiles. For the certain datasets, these limits are very narrow and all together indicated by a horizontal line with a colored circle on background. For a narrow distribution of exchange bonds ($\sigma = 0.05$), bits tend to the single-domain state independently on their diameter. Growth of σ lead to the reduction of the total area occupied by the "up" domains down to about 60% for the bit sizes considered in simulations. Statistics is collected based on 10 simulations per each set of parameters.
